# Influence of Plasticizers on Cross-Linking Process, Morphology, and Properties of Lignosulfonate-Filled Rubber Compounds

**DOI:** 10.3390/polym17030393

**Published:** 2025-02-01

**Authors:** Ján Kruželák, Michaela Džuganová, Andrea Kvasničáková, Jozef Preťo, Ján Hronkovič, Ivan Hudec

**Affiliations:** 1 Department of Plastics, Rubber and Fibres, Faculty of Chemical and Food Technology, Slovak University of Technology in Bratislava, Radlinského 9, 812 37 Bratislava, Slovakiaivan.hudec@stuba.sk (I.H.); 2 VIPO a.s., Gen. Svobodu 1069/4, 958 01 Partizánske, Slovakia

**Keywords:** calcium lignosulfonate, plasticizers, rubber, cross-linking, morphology, properties

## Abstract

Calcium lignosulfonate as a biopolymer component was incorporated into acrylonitrile butadiene rubber in the amount—50 phr. Four low-molecular organic molecules—1,4-butanediol, ethylene glycol, and two glycerols with different purity—were used as plasticizers. They were applied in rubber compounds in the amount ranging from 5 to 30 phr. The influence of the plasticizers on the curing process, cross-link density, morphology, and physical–mechanical properties was investigated. The blooming of plasticizers was also under observation. The results revealed that the application of plasticizers resulted in the deceleration of the curing kinetics and reduction in torque increments, pointing to the decrease in the rubber compounds’ viscosity. This was subsequently confirmed by the rheological measurements. The plasticizers softened the rubber matrix as well as the biopolymer filler. The higher the polarity of the plasticizer, the higher the plasticizing effect on lignosulfonate. The plasticizing effect increased in the following order: 1,4-butanediol < ethylene glycol < glycerols. Softened lignosulfonate formed small, soft filler-like domains well distributed within the rubber matrix. Good compatibility and adhesion between the rubber and the biopolymer on their interface was observed, leading to the enhancement in the tensile characteristics of the vulcanizates plasticized with ethylene glycol and glycerols.

## 1. Introduction

Traditional elastomer materials have found application in many areas of industrial and social life and have significantly improved the quality of human life. Due to the increasing demand for polymers and rubber-based products, and due to the fact that polymers originate from petroleum-based sources, there has been an increasing concern arising from the negative impact of these materials on the environment. The lack of degradability, safety, and health problems, as well as the accumulation of polymer waste in the surroundings, has led to awareness towards the production of sustainable and ecofriendly material alternatives [1].

One way for the production of greener and more sustainable polymer products is the utilization of natural macromolecules as fillers or components in polymer matrices. Starch, lignin, or cellulose represent favorable raw materials for the fabrication of multifunctional, biodegradable polymers [2,3,4,5]. Lignin is the second most spread bioorganic material on Earth following cellulose. It exhibits an amorphous, highly branched aromatic structure consisting of guaiacyl, syringyl, and p-hydroxyphenyl units. In addition, it contains several functional groups, such as methoxyl, carboxyl, aliphatic, or phenolic hydroxyls [6,7]. Lignin exhibits impressive properties, including high mechanical stability, good physical–mechanical characteristics, and adhesive, antioxidant, anti-UV, or antimicrobial properties [5,8,9,10,11,12]. Moreover, it has high thermal stability and diverse possible modifications. The annual production of lignin is about 50 million tons, coming mainly from the paper and pulp industry [13]. Despite that, only 2% of industrially obtained lignin is commercially utilized to produce value-added products. High availability, environmental friendliness, biodegradability, and reinforcing capability make it a suitable candidate as a filler or component for rubber compounds to form novel green composite materials. Through the augmentation of polar groups, enhanced electrostatic attraction, and hydrogen bonding, it can form a conjoined network with some polar polymers [13,14]. However, due to the formation of strong intramolecular interactions between the macromolecular chains, its compatibility and adhesion with non-polar polymers is usually weak. Therefore, the incorporation of lignin in its original, unmodified form into polymer compounds usually results in the deterioration in the physical–mechanical and utility properties of the final materials.

To improve the properties of lignin-based rubber composites, the combination of the biopolymer with traditional fillers used in rubber technologies has been performed [15,16,17]. Bahl et al., in their study [18], investigated hybrid fillers based on carbon black and lignin on the viscoelastic dissipation and physical–mechanical properties of rubber compounds based on styrene–butadiene rubber. The results revealed that the tensile strength of the composite with hybrid filler in the lignin to carbon black ratio of 10 to 90 (30 phr) was close to that of the equivalent composite filled only with carbon black (30 phr). The study demonstrated the formation of II–II interactions between both fillers, and the process subdues the networking of carbon black by filling the space between carbon black particles and the formation of a lignin coating layer. The experimental work performed by Yu et al. [19] demonstrated that a composite containing 30 phr of silica and 20 phr of lignin in a hybrid filler was found to have the optimal overall physical–mechanical characteristics. Simultaneously, this composite possessed low rolling resistance and high wet grip properties, which seem to be very promising for green tire technology.

Another approach for improving the utility properties of rubber products is the modification of the biopolymer to enhance the adhesion on the interfacial region between lignin and the rubber matrix. Ferruti et al. [20] performed the methacrylation of lignin through mechanochemistry, resulting in an additional functionalization for phenolic and aliphatic hydroxyl groups. The modified lignin was coprecipitated with natural rubber latex and tested as a reinforcing agent in model sidewall and tread compounds. It was revealed that modified lignin was covalently bonded to rubber chains during the curing process. This resulted in a higher compatibilization, dispersion, and stiffness when compared to the composite filled with unmodified lignin. Shorey et al. [21] used a novel silylation reaction to modify kraft lignin to increase its hydrophobicity and subsequent dispersion in natural rubber. The incorporation of 5 wt.% modified lignin into a natural rubber matrix resulted in an over 44% increase in tensile strength. The increase in elastic moduli and Payne effect intensity was observed at a higher biopolymer content. Hait, with his collective [22], introduced “in-situ surface modification of lignin utilizing a thermo-chemo-mechanical approach” during which they incorporated kraft lignin and a surface modifier, (3-aminopropyl) triethoxysilane, into rubber compounds. The resulting composite demonstrated significant improvement in tensile strength (around 15 MPa) when compared to the gum vulcanizate (1–2 MPa). Simultaneously, the composite showed a higher degree of reinforcement than a passenger car tire model compound comprised of silica and a coupling agent—polysulfide-based silane—at a similar filler loading. Sekar et al. [23] investigated lignin, which underwent hydrothermal treatment as a filler for butadiene and styrene–butadiene rubber compounds. Bis(triethoxysilylpropyl)tetrasulfide silane was used as a coupling agent for the “in situ” surface compatibilization of lignin and for an improvement in adhesion between the filler and the rubber. The outputs revealed that modified lignin exhibited semi-reinforcing behavior. Aini et al. [24], in their work, used hydroxymethylation to improve the adhesion between lignin and natural rubber-/polybutadiene rubber-based compounds. It was shown that hydroxymethylation resulted in a much higher compatibility on the filler–rubber interface and subsequent improvement in mechanical properties (tensile properties, compression set, and hardness) of composites.

The various modification techniques to improve the dispersion of lignin in rubber matrices and compatibility between both components have been described in several other studies, too [25,26,27,28,29]. The results have demonstrated very promising prospects and pointed to the high application potential of lignin into rubber compounds. However, a lot of modification procedures are time-consuming and require extra expenses. Sometimes, it is worth using a simple solution to improve the homogeneity and compatibility between the components of rubber blends, mainly for those fabricated on an industrial scale. To improve the adhesion and homogeneity between the rubber and the biopolymer in the current work, four low-molecular-weight polar plasticizers were used, namely 1,4-butanediol, ethylene glycol, and two types of glycerol (with 99% purity and in the form of 86% water solution). Those plasticizers are highly available and cost-effective. They have been chosen due to their polarity, as calcium lignosulfonate and NBR are polar materials. It is well known that low-molecular-weight hydrophilic substances are suitable plasticizers for polar-based rubber formulations. The main aim of using the plasticizers was to improve the dispersion of the biopolymer within the rubber matrix and to enhance the compatibility between both components.

## 2. Experimental Section

### 2.1. Materials

Acrylonitrile–butadiene rubber NBR, type SKN 3345 (acrylonitrile content 31–35%), was supplied from Sibur International, Russia. Calcium lignosulfonate (Borrement CA120), provided by Borregaard Deutschland GmbH, Karlsruhe, Germany, was used as a biopolymer component and was dosed to the rubber compounds in a constant amount—50 phr. The average molecular weight of lignosulfonate was 24,000 g·mol^−1^. In addition to carbon (46.63 wt.%), lignosulfonate consisted of hydrogen (5.35 wt.%), sulfur (5.62 wt.%), and nitrogen (0.14 wt.%). The amount of hydroxyl groups was equal to 1.56 wt.%. 1,4-butanediol (99%), ethylene glycol (99%), glycerol (99%), and glycerol in the form of 86% water solution (glycerol 86%) were applied into rubber formulations as plasticizers in a concentration scale ranging from 5 to 30 phr. All plasticizers were delivered from Sigma-Aldrich, St. Louis, MO, USA. For cross-linking of rubber compounds, a sulfur vulcanization system was used. The activators, stearic acid (2 phr) and zinc oxide (3 phr), were supplied from Slovlak, Košeca, Slovak Republic. Accelerators, N-cyclohexyl-2-benzothiazole sulfenamide CBS (1.5 phr) and sulfur (3 phr), as curing agents, were provided by Vegum, Dolné Vestenice, Slovak Republic. Phr stands for parts per hundred rubber.

### 2.2. Methods

#### 2.2.1. Fabrication and Curing of the Compounds

The rubber formulations were fabricated by using a Brabender laboratory kneading machine (Brabender GmbH & Co. KG, Duisburg, Germany) in two mixing steps at a temperature of 90 °C and 55 rpm. The fabrication process is summarized in Table 1. After each step, the compounds were processed into thin sheets in a two-roll mill.

The curing process was carried out using a Fontijne hydraulic press (Fontijne, Vlaardingen, The Netherlands) following the optimum cure time of each rubber compound. The curing temperature was 170 °C and the press was 15 MPa. After curing, thin sheets with dimensions of 15 × 15 cm^2^ and a thickness of 2 mm were obtained.

#### 2.2.2. Curing Characteristics

Curing isotherms were measured in an MDR 2000 oscillatory rheometer (Alpha Technologies, Akron, OH, USA). From them, the curing characteristics were determined as follows:

*M_L_*—minimum torque (dN·m);
*M_H_*—maximum torque (dN·m);
Δ*M* (dN·m)—torque difference, the difference between *M_H_* and *M_L_*;
*t_c_*_90_ (min)—optimum curing time;
*t_s_*_1_ (min)—scorch time;
*R* (dN.m.min^−1^)—curing rate, defined as follows:

(1) $$R=\frac{M_{c90}-M_{s1}}{t_{c90}-t_{s1}}$$

*M_c_*_90_ (dN.m)—torque at *t_c_*_90_;
*M_s_*_1_ (dN.m)—torque at *t_s_*_1_.

#### 2.2.3. Cross-Link Density

To calculate the cross-link density *ν*, dried and weighted samples were immersed in xylene, in which they swelled within time. The samples were taken out from the solvent every hour, wiped out of solvent, and weighed. When the weight of the samples was constant, equilibrium swelling was reached and used for the determination of the cross-link density using the Krause-modified Flory–Rehner equation [30]:

(2) $$\nu_{ch}=-\frac{V_{r0}}{V_{S}}\frac{\ln(1-V_{r})+V_{r}+\chi {V_{r}}^{2}}{{V_{r}}^{1/3}{V_{r0}}^{2/3}-0.5V_{r}}$$

*ν_ch_*—cross-link density (mol·cm^−3^);
*V_r_*_0_—volume fraction of rubber in equilibrium swelling sample of vulcanizate in absence of fillers;
*V_r_*—volume fraction of rubber in equilibrium swelling sample of filled vulcanizate;
*V_S_*—molar volume of solvent (for xylene = 123.45 cm^3^·mol^−1^);
*χ*—Huggins interaction parameter (for measuring conditions *χ* = 0.5316).

#### 2.2.4. Rheological Measurements

Dynamic complex viscosity was investigated using RPA 2000 (Alpha Technologies, Akron, OH, USA). The frequency was set up to 0.2 Hz, the temperature of the measurement was 90 °C, the strain amplitude changed from 0.15 to 700%.

#### 2.2.5. Physical–Mechanical Characteristics

The tensile tests were performed according to valid technical standards using a Zwick Roell/Z 2.5 tearing equipment (Zwick GmbH & Co. KG, Ulm, Germany). The cross-head speed was set up to 500 mm.min^−1^ with a gauge length of 25 mm. The dumbbell-shaped specimens (width 6.4 mm, length 80 mm) were cut with a special knife from a 2 mm thick cured rubber plate. The hardness in Shore A was measured by using a durometer.

#### 2.2.6. Microscopic Analysis

The surface morphology was investigated using a JEOL JSM-7500F scanning electron microscope SEM (Jeol Ltd., Tokyo, Japan). The samples were frozen in liquid nitrogen and fractured into small fragments. The uncovered fracture surfaces were coated with a thin gold layer. The accelerated voltage of the microscope ranged from 0.1 kV to 30 kV. The resolution was 1.0 nm at 15 kV and 1.4 nm at 1 kV. SEM images were captured by CCD-Camera EDS (Oxford INCA X-ACT, Oxford Instruments, Abingdon, UK).

#### 2.2.7. Blooming Test

To observe the blooming of plasticizers, small samples with dimensions of 1 × 1 cm^2^ were washed with ethanol, dried, and weighed one day after the curing process. Subsequently, the samples were hung on the hook and placed in the oven at 70 °C. The samples were taken out of the oven, washed with ethanol, and weighed after 2, 5, and 24 h. The blooming was calculated as the weight loss of the samples related to the surface of the test specimens:

(3) $$WL=\frac{m_{1}-m_{2}}{S}$$

*WL*—weight loss (g·m^−2^);
*m*_1_—weight of the sample at the beginning of the test (g);
*m*_2_—weight of the sample at the end of the test (g);
*S*—surface area of the sample (S = 1 cm^2^).

## 3. Results and Discussion

### 3.1. Curing Process and Cross-Link Density

**Figure 1:**
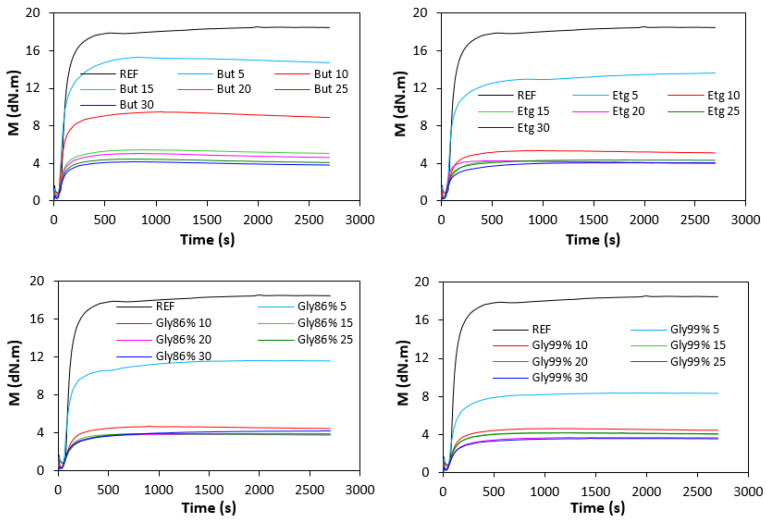
Vulcanization isotherms of the compounds.

**Figure 2:**
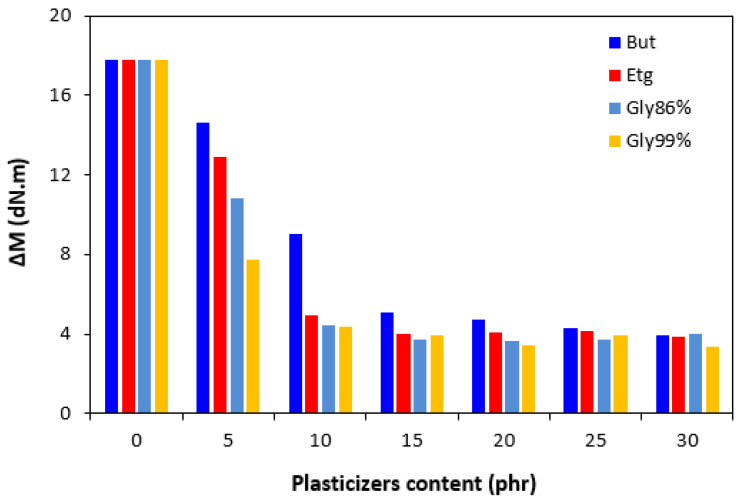
Influence of plasticizer content on torque difference Δ*M* in the compounds.

The curing characteristics of the rubber compounds were evaluated from the corresponding curing isotherms measured by the MDR 2000 rheometer (Figure 1). It becomes apparent from them that both the minimum *M_L_* but mainly the maximum *M_H_* torque showed a significant decreasing trend with the increasing amounts of plasticizers. The highest *M_L_* and *M_H_* was exhibited by the reference, plasticizer-free rubber compound, while the lowest values of both characteristics were demonstrated by the rubber compounds with the maximum plasticizer content. The decrease in the minimum torque relates to the decrease in the viscosity before the curing process begins, while the decline in the maximum torque refers to the decrease in the viscosity and cross-link density of the cured compounds. The decrease in the *M_H_* and *M_L_* was subsequently reflected in the decrease in the torque difference (Δ*M* = *M_H_* − *M_L_*), as seen in Figure 2. The *M_H_*, *M_L_*, and Δ*M* were significantly lowered up to 10 phr of plasticizers, but then there was only a small change with the next increase in the plasticizer content. The type of plasticizer had no significant influence on the minimum and maximum torque as well as on the torque difference. However, it seems that the highest Δ*M* was demonstrated by the vulcanizates with applied 1,4-butanediol, mainly at lower amounts of plasticizer. The decrease in the torque values by the application of plasticizers points to their softening effect on rubber compounds and lowering of the viscosity.

**Figure 3:**
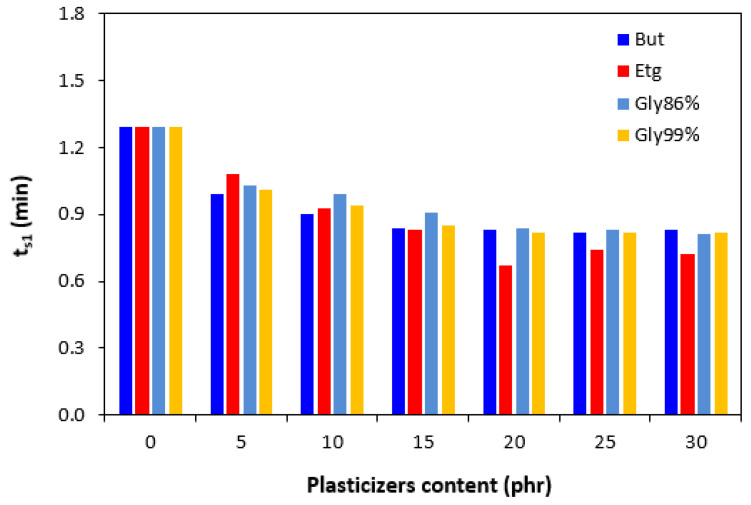
Influence of plasticizer content on scorch time *t_s_*_1_ of the compounds.

**Figure 4:**
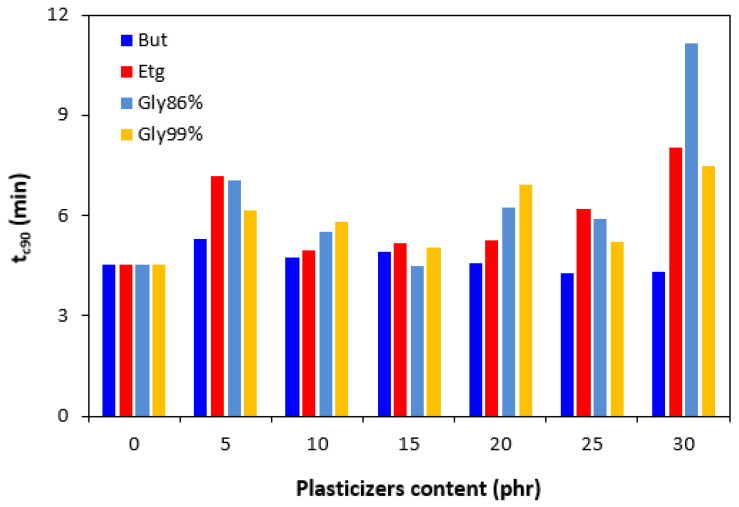
Influence of plasticizer content on optimum cure time *t_c_*_90_ of the compounds.

**Figure 5:**
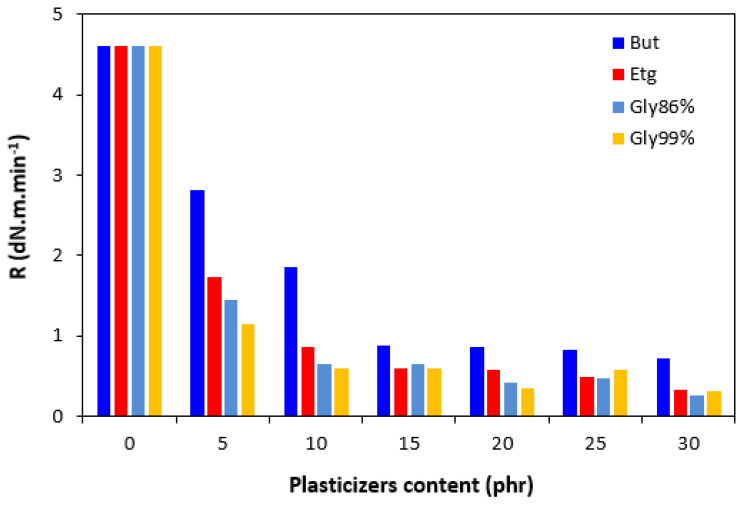
Influence of plasticizer content on cure rate *R* of the compounds.

The presence of plasticizers in the rubber compounds resulted in a small decrease in the scorch time *t_s_*_1_ (Figure 3). On the other hand, by the introduction of 5 phr plasticizers, the optimum cure time *t_c_*_90_ was prolonged when compared to the reference (Figure 4). Then, no clear influence of the plasticizer content was observed on the optimum cure time. In most cases, the *t_c_*_90_ was significantly prolonged at the maximum plasticizer content. The analysis of the curing process kinetics was also observed by the evaluation of the cure rate *R*. From Figure 5, it becomes apparent that the highest cure rate demonstrated was for the reference rubber compound. The higher the amount of plasticizers, the lower the cure rate. When considering the type of plasticizer, the highest cure rate demonstrated was for the compounds plasticized with 1,4-butanediol. The most significant decrease in the cure rate was recorded at a low plasticizer content. The higher the amount of plasticizers, the lower the differences in the cure rate. A possible explanation for the decrease in the curing kinetics might be the polarity of plasticizers, due to which they can absorb or dilute the additives of curing systems and make them ineffective during the vulcanization process [31].

### 3.2. Rheological Measurement

**Figure 6:**
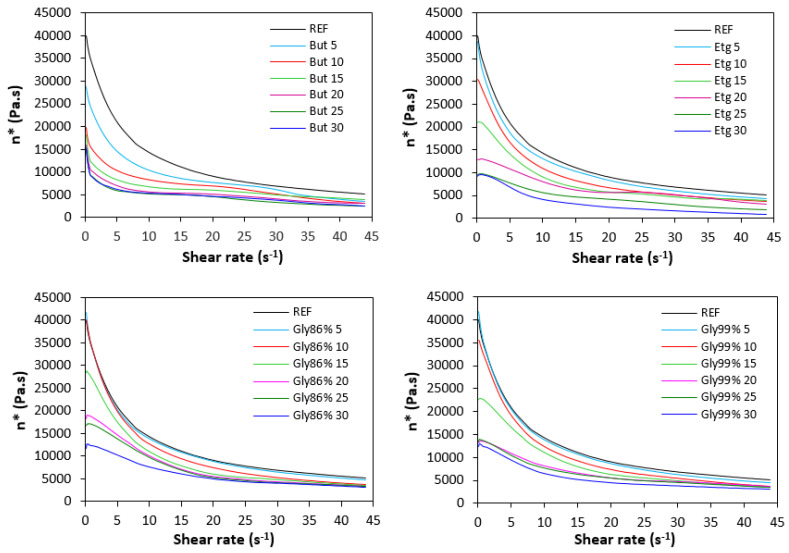
Dynamic complex viscosity η* of the compounds depending on shear rate.

The shear rate dependences of the dynamic complex viscosity η* are depicted in Figure 6. As shown, the highest viscosity in the whole range of the shear rate was manifested by the reference plasticizer-free sample. By the application of plasticizers, the viscosity decreased. The samples with maximum contents of plasticizers demonstrated the lowest viscosity. The differences in complex viscosities are more prone at lower shear rates. The higher the shear rate, the lower the differences in viscosities depending on the plasticizer content. The achieved outputs are in line with the data summarized in a previous section. Small molecules of plasticizers enter the intermolecular space between the chains and disrupt inter- and intramolecular physical interactions and entanglements. This results in a reduction in internal friction and increase in the chain segments’ mobility. Simultaneously, plasticizers soften calcium lignosulfonate. They enable better dispersion and distribution of the biopolymer within the rubber matrix, which contributes to the lowering of the viscosity, too. The effect of each plasticizer on the biopolymer filler with respect to the final properties of vulcanizates is more closely discussed in the morphology section.

### 3.3. Cross-Link Density and Physical–Mechanical Properties

**Figure 7:**
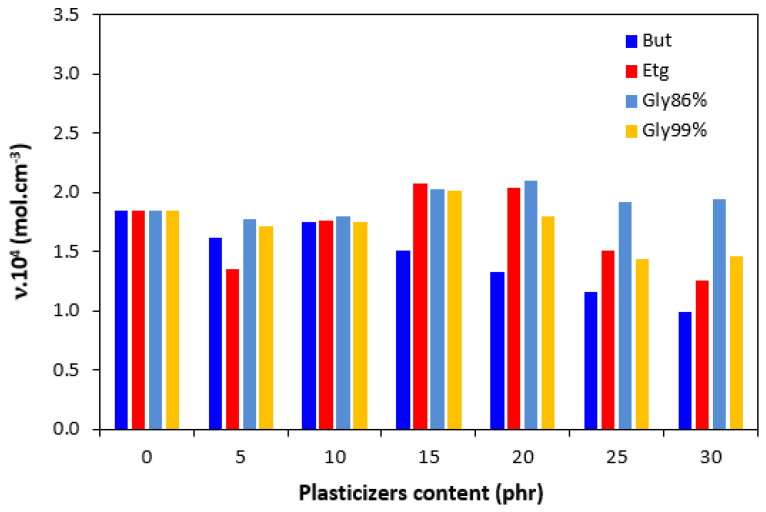
Influence of plasticizer content on cross-link density *υ* of vulcanizates.

The cross-link density *ν* is an important structural characteristic that influences the properties of the vulcanized rubber compounds. The cross-linking degree of vulcanizates depending on the type and amount of plasticizer is depicted in Figure 7. It becomes apparent that the application of 1,4-butanediol resulted in the decrease in the cross-linking degree, which is evident mainly at higher amounts of plasticizer. The influence of ethylene glycol and glycerols on the cross-link density was low. It can be stated that the cross-linking degree passed over a slight maximum at 15 and 20 phr of plasticizers and slightly dropped down. The differences in *ν* became more evident at higher amounts of plasticizers (20–30 phr). In that case, the highest cross-link density was exhibited by the vulcanizates plasticized with glycerol 86%.

**Figure 8:**
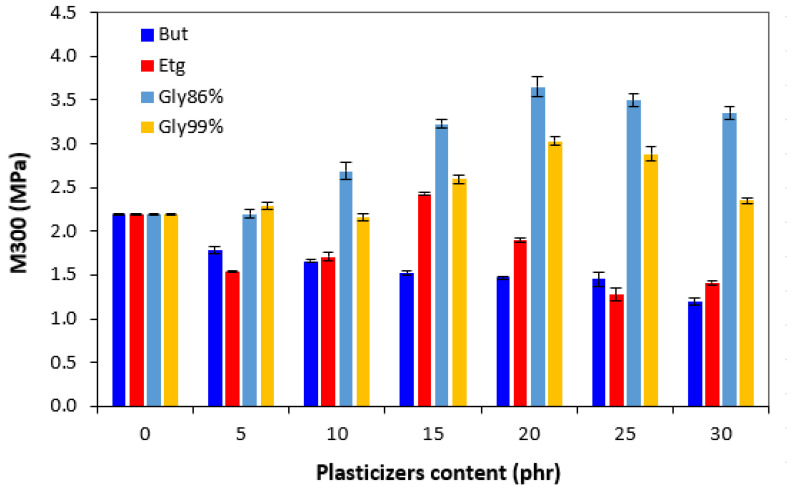
Influence of plasticizer content on modulus M300 of vulcanizates.

Looking at Figure 8, one can see a correlation between the modulus M300 and cross-link density. As the lowest degree of cross-linking was exhibited by vulcanizates with applied 1,4-butanediol, these systems were also found to have the lowest modulus. The M300 showed a decreasing trend with an increasing amount of plasticizer, again following the trend of the cross-link density. On the other hand, the highest cross-link density of the materials softened with glycerol 86% was reflected in the highest modulus. The modulus of vulcanizates plasticized with 1,4-butanediol and ethylene glycol decreased when compared to the reference. On the other hand, the opposite tendency was recorded for vulcanizates with both glycerols. The M300 of the equivalent vulcanizates passed over a maximum at 15–20 phr of the plasticizers.

**Figure 9:**
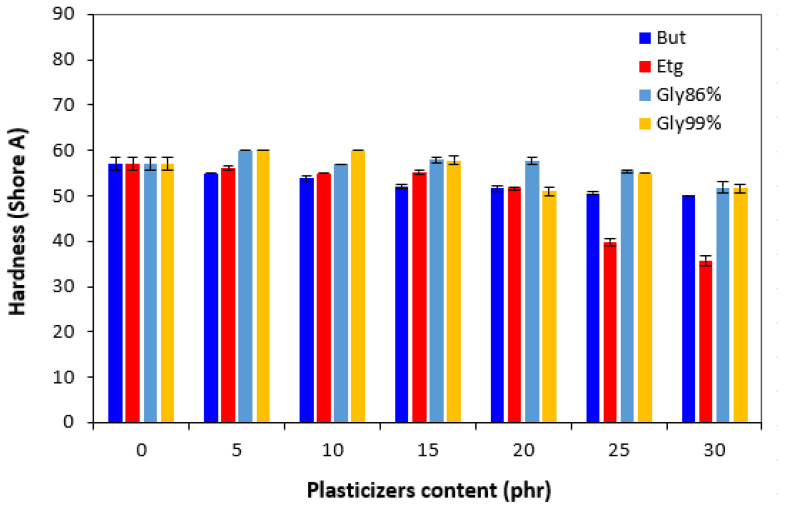
Influence of plasticizer content on hardness of vulcanizates.

**Figure 10:**
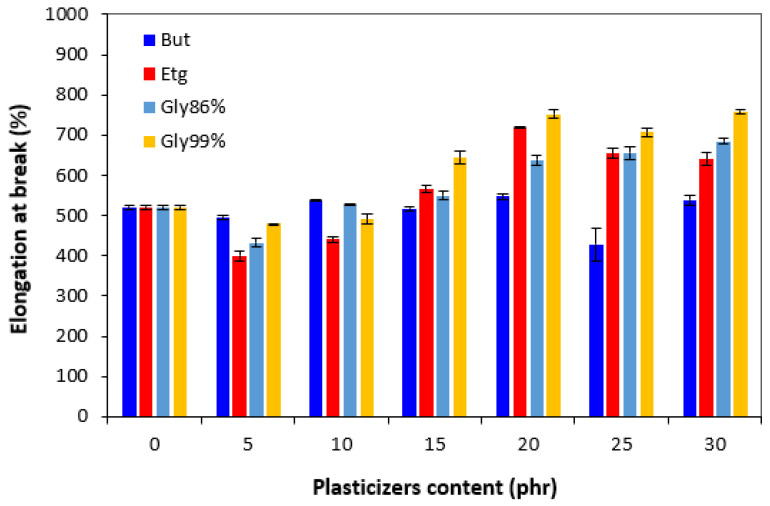
Influence of plasticizer content on elongation at break of vulcanizates.

The hardness did not change very much up to 10–15 phr of plasticizer; then, the decrease was recorded for vulcanizates with higher amounts of plasticizers (Figure 9). However, the decrease was not very significant. From Figure 10, it is shown that in comparison with the reference, the elongation at break first slightly decreased by the incorporation of 5 phr plasticizers. The elongation at break of vulcanizates with 1,4-butanediol fluctuated only in a low experimental value range, almost independently of the content of the plasticizer and was very similar to that of the reference. The elongation at break of the vulcanizates with applied ethylene glycol and both glycerols showed an increasing trend and reached a maximum at 20 phr of plasticizers. The decrease in the hardness and the increase in the elongation at break can be attributed to the softening effect of plasticizers on rubber compounds, weakening of intramolecular interactions, and thus increase in rubber chains’ elasticity and mobility.

**Figure 11:**
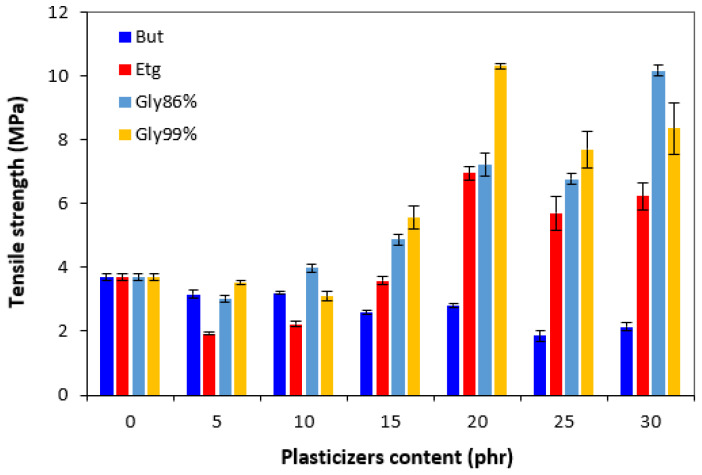
Influence of plasticizer content on tensile strength of vulcanizates.

Similarly to the elongation at break, the tensile strength first decreased by the application of 5 phr plasticizers (Figure 11). Subsequently, the increase in the tensile strength was recorded (with exclusion of the compounds with 1,4-butanediol). The tensile strength of the compound with a maximum content of glycerol 86% was almost threefold higher when compared to the reference (the tensile strength increased from 3.7 MPa for the reference up to over 10 MPa for the vulcanizate with 30 phr of glycerol). By the application of glycerol 99%, the maximum tensile strength was reached at 20 phr of the plasticizer (again over 10 MPa). Similarly, vulcanizates plasticized with ethylene glycol reached a maximum tensile strength at 20 phr of the plasticizer. When compared to the reference, the application of 20 phr ethylene glycol resulted in an increase in the tensile strength up to over 6 MPa. By contrast, the tensile strength of the vulcanizates plasticized with 1,4-butanediol decreased. As shown in Figure 11, the higher the amount of 1,4-butanediol, the lower the tensile strength. Based upon the obtained results, it can be concluded that the presence of plasticizers in rubber formulations (except for 1,4-butanediol) resulted in an increase in the tensile characteristics. However, the enhancement in the tensile strength and elongation at break was observed at higher amounts of plasticizers. It also becomes evident that the highest tensile strength was demonstrated by the vulcanizates plasticized with both glycerols.

The effect of plasticizers on the properties of lignin-based polymer composites was also studied in some other works. For example, Datta and Parcheta [32] made a comparative study on the properties of kraft lignin natural rubber-based composites softened with different plasticizers. They used glycerolysate as the semi-product of the chemical recycling of polyurethanes as a novel plasticizer and compared it with commonly used naphthenic oil. The results revealed that glycerolysate-based composites exhibited comparable or even better properties when compared to those based on commonly used plasticizers based on naphthenic oil. The best properties were exhibited by the composite containing 10 phr of lignin, though the tensile strength was still lower when compared to the reference. Dominici et al. [33], in their study, revealed that the addition of environmentally friendly plasticizers obtained from vegetable oils, maleinized, and epoxidized linseed oil resulted in an increase in the mechanical properties of lignin/natural fiber composites, such as the flexural strength (by 49%) and tensile strength (by 43%). The results also showed that the added plasticizers improved the thermal stability of the neat rubber matrix. In the study performed by Bartolome et al. [34], the use of plasticizers such as polyethylene glycol with different molecular weights, malic acid, xylitol, glycerol, and sorbitol into lignosulfonate-based polymer materials prevented brittleness and improved the tensile characteristics of the final materials. In general, it can be concluded that the best performing were plasticizers based on polyethylene glycol, improving both the elongation at break in more than 50% and tensile strength in more than 40 MPa. Materials with applied glycerol demonstrated poor tensile strength but the highest elongation at break (over 100%). Malic acid was the worst-performing plasticizer as it caused the stiffness and rigidity of the polymers. The study also indicated that plasticizers modify the strong intra- and inter hydrogen interactions in lignosulfonate and improved the elasticity and flexibility of the final materials. The works point to the prospective application of plasticizers in lignin-based polymer composites, which can be used as suitable modifiers of the final properties.

### 3.4. Morphology

**Figure 12:**
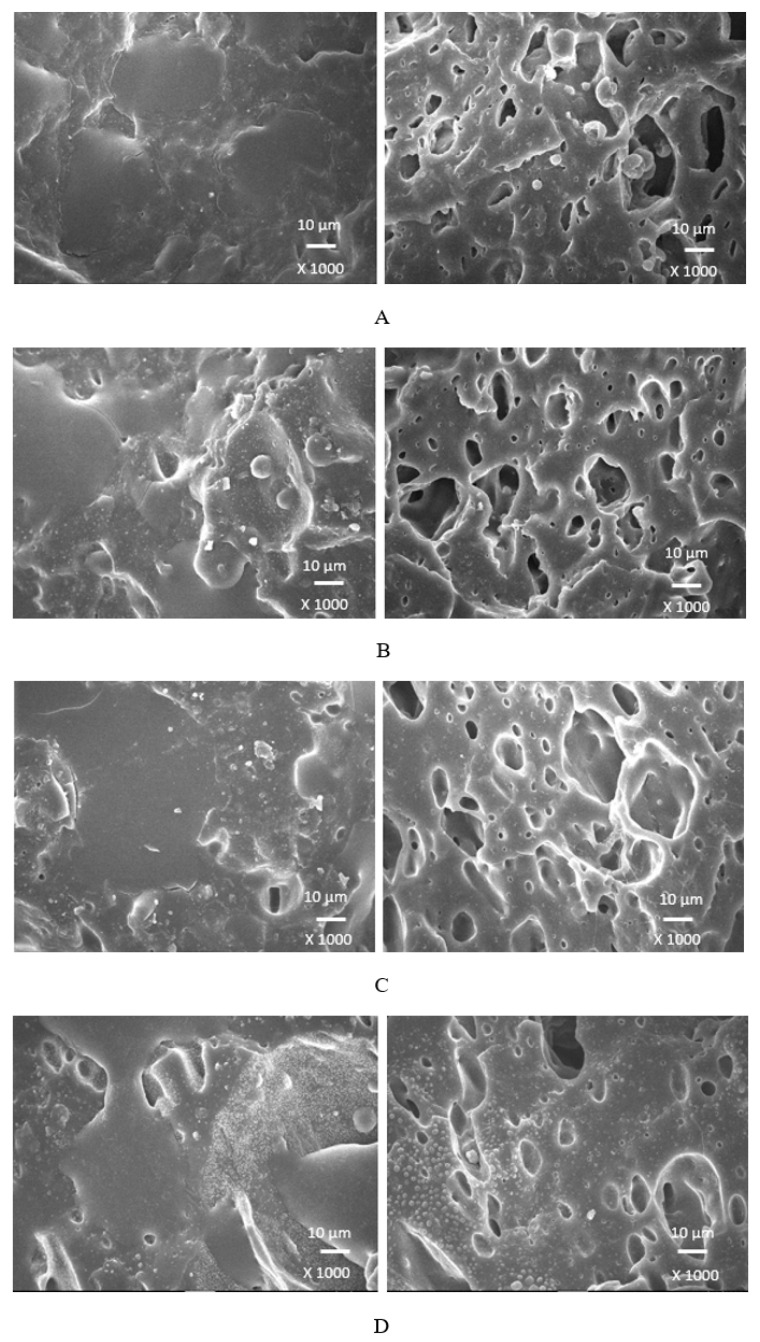
SEM images of vulcanizates with applied 1,4-butanediol: (**A**) reference plasticizer-free vulcanizate, (**B**) vulcanizate with 10 phr of 1,4-butanediol, (**C**) vulcanizate with 20 phr of 1,4-butanediol, (**D**) vulcanizate with 30 phr of 1,4-butanediol. SEM images of vulcanizates before washing in hot water are presented on the left side, while SEM images of vulcanizates after washing in boiling water are presented on the right side.

**Figure 13:**
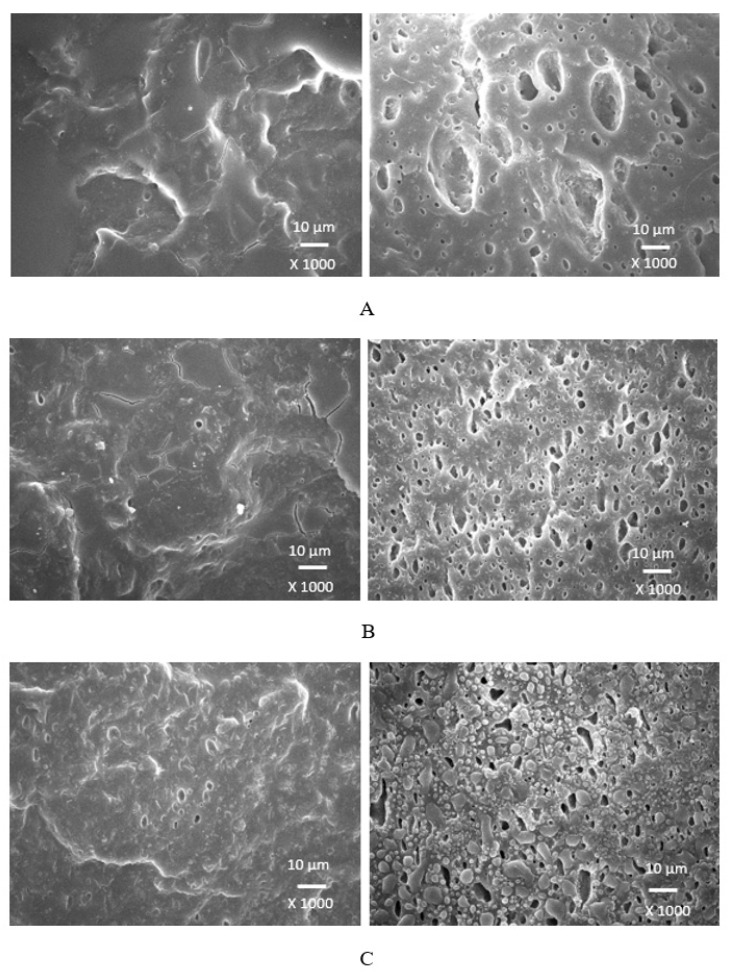
SEM images of vulcanizates with applied ethylene glycol: (**A**) vulcanizate with 10 phr of ethylene glycol, (**B**) vulcanizate with 20 phr of ethylene glycol, (**C**) vulcanizate with 30 phr of ethylene glycol. SEM images of vulcanizates before washing in hot water are presented on the left side, while SEM images of vulcanizates after washing in boiling water are presented on the right side.

**Figure 14:**
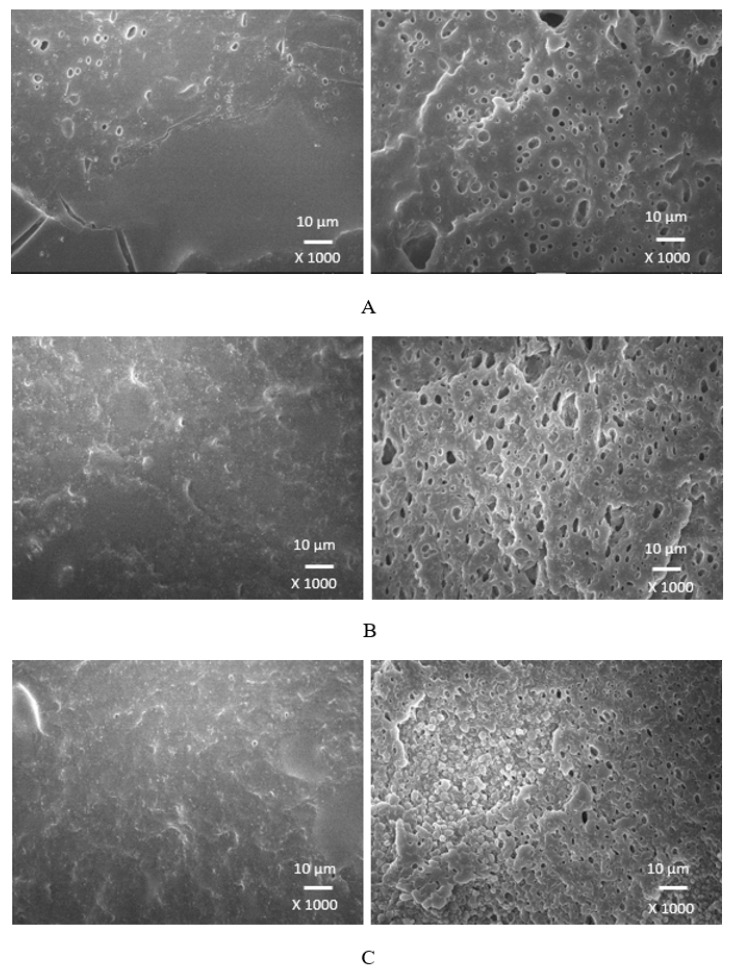
SEM images of vulcanizates with applied glycerol 86%: (**A**) vulcanizate with 10 phr of glycerol 86%, (**B**) vulcanizate with 20 phr of glycerol 86%, (**C**) vulcanizate with 30 phr of glycerol 86%. SEM images of vulcanizates before washing in hot water are presented on the left side, while SEM images of vulcanizates after washing in boiling water are presented on the right side.

**Figure 15:**
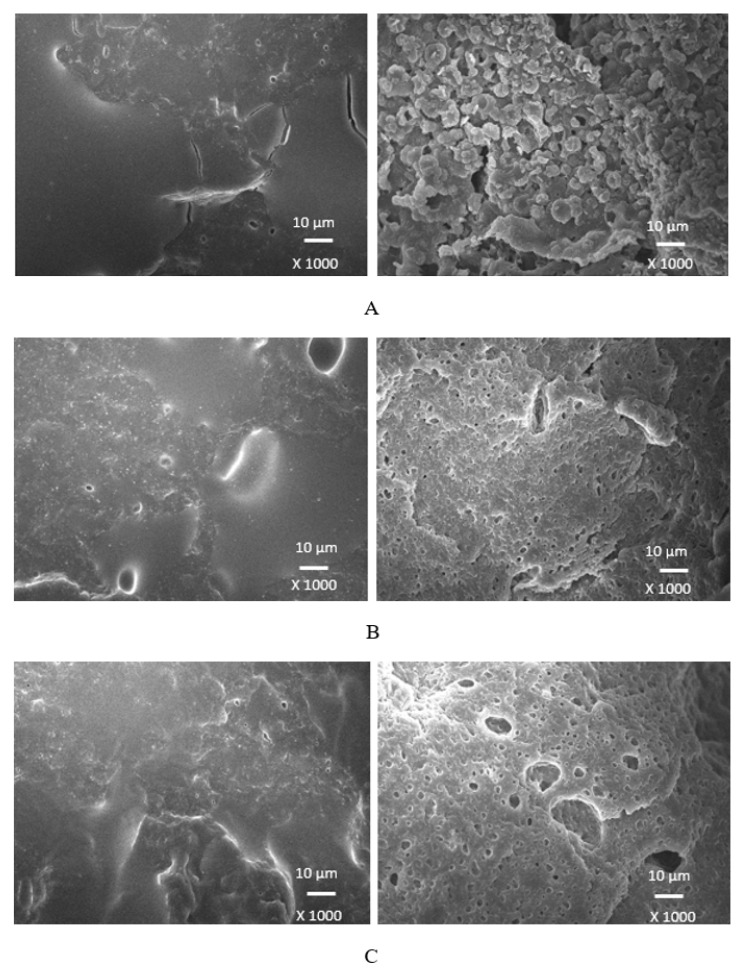
SEM images of vulcanizates with applied glycerol 99%: (**A**) vulcanizate with 10 phr of glycerol 99%, (**B**) vulcanizate with 20 phr of glycerol 99%, (**C**) vulcanizate with 30 phr of glycerol 99%. SEM images of vulcanizates before washing in hot water are presented on the left side, while SEM images of vulcanizates after washing in boiling water are presented on the right side.

Scanning electron microscopy was employed to analyze the surface structure and morphology of vulcanizates. The samples were first frozen in liquid nitrogen and then fractured. The uncovered surfaces were subjected to microscopic analysis and the SEM images of the vulcanizates are presented in Figure 12, Figure 13, Figure 14 and Figure 15 on the left side. To better evaluate the dispersion and distribution of the biopolymer throughout the rubber matrix, SEM analysis was also performed after the samples were washed in boiling water for two hours. As calcium lignosulfonate is water soluble, it was removed from the surface, unveiling the holes and cavities, which were occupied by biopolymer filler before its extraction by hot water. The SEM images of the boiling water-treated vulcanizates are presented in Figure 12, Figure 13, Figure 14 and Figure 15 on the right side. From Figure 12, it becomes apparent that lignosulfonate tended to form agglomerates in the reference sample. The same statement can also be applied for vulcanizates with lower amounts of plasticizers. The presence of agglomerates confirms that the dispersion of the biopolymer and mutual compatibility between the rubber and the filler on their interfacial region is weak. To that correspond the low physical–mechanical properties, mainly the weak tensile strength of the reference sample and vulcanizates with a lower plasticizer content. The increasing amounts of plasticizers resulted in the better dispersion and distribution of the biopolymer within the rubber matrix and better adhesion between both components. The structures of the fracture surfaces look smoother and more compact, without any apparent structural defects. The used plasticizers are hydrophilic, low-molecular organic molecules that plasticize both the rubber matrix and calcium lignosulfonate. This leads to a decrease in the viscosity of the rubber compounds, as previously confirmed by the rheological measurements. The viscosity of the filler and the rubber became closer, which resulted in the better dispersion and distribution of the biopolymer. The improvement in the compatibility and adhesion on the filler–rubber interface was achieved. The enhancement in the dispersion and distribution of the biopolymer within the rubber matrix is clearly observed from the SEM images of the vulcanizates washed in boiling water (Figure 12, Figure 13, Figure 14 and Figure 15 on the right). It becomes obvious that the sizes of the agglomerates were reduced, and lignosulfonate formed smaller and softer domains. The softer domains with a lower hardness and rigidity deform much more easily, when compared to the stiff filler agglomerates, which act as stress concentrators upon deformation strains. This causes a decrease in the mechanical properties of the final rubber materials. On the other hand, small domains with high deformability behave as particles of reinforcing fillers, contributing to the increase in the tensile behavior of rubber systems [35,36,37].

When considering the type of plasticizer, it can be deduced that the least homogeneous structure with big filler domains was demonstrated by the vulcanizates with applied 1,4-butanediol (Figure 12). 1,4-butanediol contains two hydroxyl groups situated at the ends of a longer hydrocarbon chain, which results in the lowest relative polarity from the tested plasticizers. Thus, it can be deduced that it has the lowest influence on the softening of the biopolymer. The achieved outputs confirmed the presumption showing that lignosulfonate formed big agglomerates and weak adhesion on the filler–rubber interface was observed. This resulted in the deterioration in the physical–mechanical properties of the vulcanizates. Ethylene glycol also has two hydroxyl groups but attached to a shorter hydrocarbon chain. Thus, it exhibits a higher relative polarity and higher plasticizing effect on calcium lignosulfonate. A better dispersion and distribution of the biopolymer was achieved (Figure 13). The SEM analysis as well as the determined physical–mechanical properties of the vulcanizates are in line with this statement. The most homogeneous and compact structure was manifested by the vulcanizates plasticized with both glycerols (Figure 14 and Figure 15). The molecule of glycerol contains three hydroxyl groups, which results in the highest polarity and the highest plasticizing effect on the biopolymer. It becomes apparent from the SEM images that lignosulfonate formed soft, small domains well distributed throughout the rubber matrix. Very good adhesion and compatibility on the filler–rubber interfacial region was also observed. The corresponding vulcanizates plasticized with glycerol exhibited the highest tensile characteristics. It can be stated that water present in glycerol also has a positive influence on the softening of lignosulfonate and contributes to its better dispersion and distribution [38].

### 3.5. Blooming of Plasticizers

The formulated rubber compounds are usually multicomponent systems, containing low-molecular substances, such as curing system additives, antidegradants, waxes, plasticizers, and other compounding additives. Some of these substances often migrate to the rubber surface and form a very thin layer on the vulcanizates. This phenomenon is called blooming. With respect to the requirements for the final properties, blooming can be a desired, but also a negative effect. For example, wax blooming is used to protect rubber articles against ozone attack by the formation of a thin coherent layer acting as a physical barrier [39]. Blooming can also be undesired, for example, the migration of ingredients sometimes causes surface tackiness, reduces the aesthetic appearance, or gives rise to poor rubber to rubber or rubber to metal adhesion [40]. Also, health and environmental aspects must be considered when blooming some additives.

**Figure 16:**
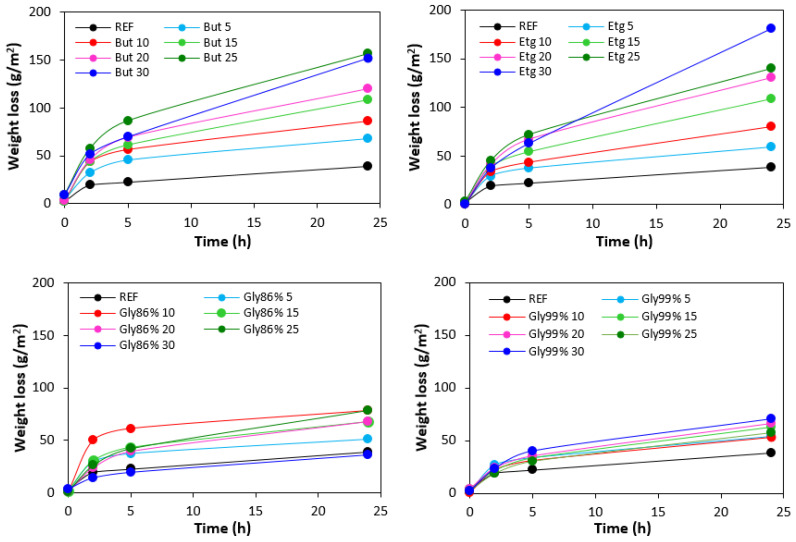
Weight loss of vulcanizates depending on time.

**Figure 17:**
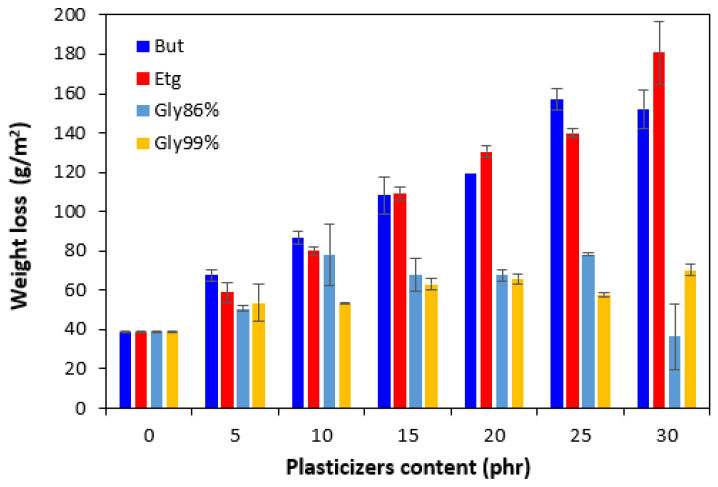
Influence of plasticizer content on weight loss of vulcanizates after 24 h.

The weight loss of the samples after being exposed to 70 °C depending on the time is graphically illustrated in Figure 16, while the weight loss depending on the type and amount of plasticizers after 24 h is presented in Figure 17. It becomes apparent from Figure 17 that the lowest weight loss was exhibited by the reference sample without plasticizer. At an initial stage, the weight loss of the reference sample might be attributed to the loss of the biopolymer, which was not well bound to the surface and was wiped out with ethanol. Then, as seen in Figure 16, there was no other significant change in the weight loss of the reference sample depending on the time. However, some increase can still be caused by the wiping out of calcium lignosulfonate from the surface layer. The higher the amount of 1,4-butanediol and ethylene glycol in the rubber formulations, the higher the weight loss, whereas the type of plasticizer played no crucial role. The weight loss of the samples with lower amounts of both glycerols was also higher when compared to the reference and was very similar to that recorded for vulcanizates with lower amounts of 1,4-butanediol and ethylene glycol. But then there was no other significant change in the weight loss with the next increase in the content of glycerols. At higher amounts of plasticizers, the weight loss of the vulcanizates plasticized with glycerols was much lower when compared to the equivalent vulcanizates softened with 1,4-butanediol and ethylene glycol.

The reason for a different specific weight loss of the vulcanizates can again be attributed to the structure of plasticizers. 1,4-butanediol and ethylene glycol are less polar due to a lower amount of hydroxyl groups. On the other hand, glycerol exhibits a higher polarity and thus higher affinity with the biopolymer. A higher amount of hydroxyl groups increases the efficiency of hydrogen bond formation between glycerol and calcium lignosulfonate. Glycerol is more physically adsorbed on the biopolymer, and thus it shows a lower blooming effect.

## 4. Conclusions

Calcium lignosulfonate-filled rubber compounds were plasticized with polar low-molecular-weight organic substances. The results revealed that the application of plasticizers resulted in the decrease in the curing kinetics and reduction in the minimum and maximum torque as well as torque difference. This pointed to the softening effect of plasticizers on rubber compounds and a reduction in the compounds’ viscosity, which was clearly confirmed from the rheological measurements. The higher the amount of plasticizers, the lower the viscosity. The reduction in internal friction between the chain segments and increase in the chains’ elasticity and mobility resulted in the decrease in the hardness and increase in the elongation at break. Simultaneously, plasticizers exhibited a softening effect on lignosulfonate. It can be stated that the higher the polarity of the plasticizer, the higher the softening effect on the biopolymer. The SEM analysis revealed that the lowest plasticizing effect on calcium lignosulfonate was exhibited by 1,4-butanediol, because of its lowest polarity. A low distribution and dispersion of the biopolymer throughout the rubber matrix and poor compatibility on the filler–rubber interface resulted in the decrease in the physical–mechanical properties of 1,4-butanediol plasticized rubber compounds. On the other hand, the most homogeneous structure was demonstrated by the vulcanizates with applied glycerols having the highest polarity. This suggests that glycerol shows the highest plasticizing effect on lignosulfonate. Softened lignosulfonate formed small, soft filler-like domains well distributed within the rubber matrix. Good compatibility on the filler–rubber interface was observed. The outlined facts are responsible for the enhancement in the tensile characteristics of the corresponding vulcanizates. A remarkable threefold increase in the tensile strength was recorded for the vulcanizates plasticized with the maximum content of glycerol 86% when compared to the reference (from less than 4 MPa for the reference sample to over 10 MPa). The tensile strength of the vulcanizate plasticized with 20 phr of glycerol 99% overcame 10 MPa, too. Similarly, the elongation at break increased by the application of ethylene glycol and both glycerols. The highest elongation at break was exhibited by vulcanizates with applied glycerol 99%. The elongation at break increased from less than 500% for the reference up to over 700% for the vulcanizate with high amounts of plasticizer. The highest polarity and highest amount of hydroxyl groups in the glycerol structure enabled the highest physical adhesion with the biopolymer. As a result, glycerol showed the lowest blooming effect.

## Figures and Tables

**Table 1:** Fabrication process of rubber compounds.

Mixing Step	Mixing Time	Mixing Material	Mixing Conditions
Step	1 min	Rubber	90 °C, 55 rpm
	1 min	ZnO + stearic acid	90 °C, 55 rpm
	4 min	Filler + plasticizer	90 °C, 55 rpm
	Cooling and homogenization of the compounds		
2. Step	4 min	Sulfur + CBS	90 °C, 55 rpm
	Cooling and homogenization of the compounds		

## Data Availability

Data are contained within the article.
